# Appointment of Dr. T. O. Edwards

**Published:** 1850-08

**Authors:** 


Dr. T. O. Edwards, of Cincinnati, has been appointed Professor of Materia Medica and Pharmacy in the Medical College of Ohio.

